# Covalent three-dimensional carbon nanotube and derived B-C-N polymorphs with superhardness and zero Poisson’s ratio

**DOI:** 10.1016/j.isci.2022.105563

**Published:** 2022-11-13

**Authors:** Shuang Chen, Meng Hu, Lingyu Liu, Yilong Pan, Penghui Li, Julong He, Jianning Ding

**Affiliations:** 1 Institute of Intelligent Flexible Mechatronics, School of Mechanical Engineering, Jiangsu University, Zhenjiang 212013, China; 2 State Key Laboratory of Metastable Materials Science and Technology, Yanshan University, Qinhuangdao 066004, China; 3 School of Materials Science and Engineering, Xihua University, Chengdu 610 039, China; 4 Institute of High Pressure Physics, School of Physical Science and Technology, Ningbo University, Ningbo 315211, China; 5 School of Mechanical Engineering, Yangzhou University, Yangzhou 225127, China

**Keywords:** Organic chemistry, materials science, mechanical processing

## Abstract

Carbon is one of the most versatile atoms and fosters a wealth of carbon allotropes with superior mechanical and electronic properties. A three-dimensional covalent carbon nanotube, named CCN, with a hexagonal honeycomb-like crystalline structure is proposed theoretically. CCN consists of *sp*^3^ bonded coaxially teamed (6,0) carbon nanotubes, and the tube walls possess intrinsic wrinkles, which trigger miraculous physical properties. The mechanical and thermal dynamic stabilities are confirmed, and molecular dynamics simulations indicate high temperature thermal stability up to 1500 K. CCN has an unusual cork-like zero Poisson’s ratio along the axial direction of the nanotubes, and the axial/radial stretching or compression rarely effects the radial/axial dimensions of the nanotubes. CCN is superhard with Vickers hardness of 82.8 GPa, matching that of cubic boron nitride. Substitution B and N atoms for C atoms result in superhard CCN-B_12_N_8_ and CCN-C_8_N_12_ with quasi-zero Poisson’s radio along both axial and radial directions.

## Highlights

• Stable covalent carbon nanotube with superhardness matching c-BN
• Abnormally wrinkled walls result in zero Poisson’s ratio property
• Radial dimensional stability under large axial tensile or compressive strain over 13%
• Derived B-C-N polymorphs exhibit superhardness and zero Poisson’s ratio

## Graphical abstract

**Figure:**
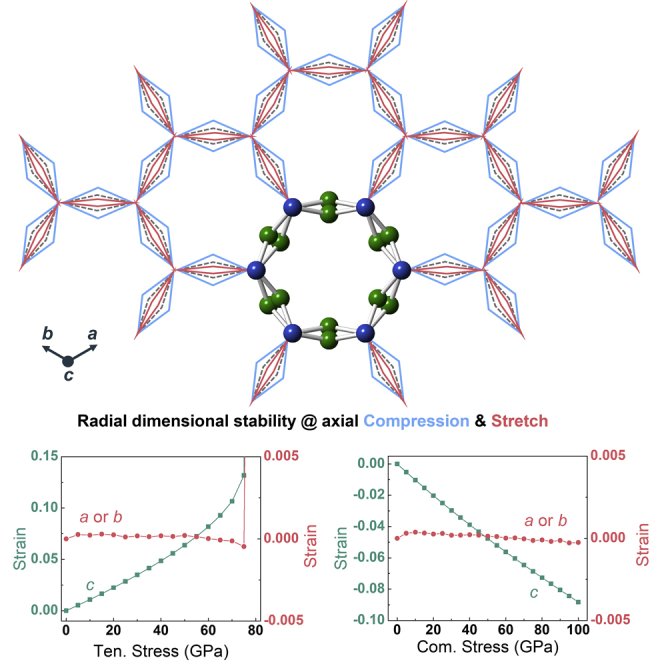

## Introduction

Carbon portfolio with striking physical and chemical properties, such as graphite, diamond, lonsdaleite, fullerene, carbon nanotubes (CNTs), graphene, graphdiyne,^1^ and amorphous carbon,^2^^,^^3^^,^^4^^,^^5^^,^^6^ have been promoted by versatile carbon atoms possessing peculiar bonding motifs of *sp*, *sp*^2^*,* and *sp*^3^ hybridization, and are playing a leading role in science and technology. Extensive efforts have been devoted to the theoretical discovery of novel carbon polymorphs^7^ exhibiting remarkable properties, including superhardness,^8^^,^^9^ ultrahigh ideal strength,^10^^,^^11^ electrical insulator,^12^ semiconductivity,^13^ metallicity,^14^^,^^15^ and superconductivity.^16^^,^^17^ Further, regulating and acquiring new carbon materials with two or more specific properties through the compounding of the carbon elements themselves is a popular research topic and a highly sought-after goal today.

The negative ratio of the transverse strain to the corresponding axial strain is defined as Poisson’s ratio. Most materials exhibit a positive Poisson’s ratio (PPR), which means the material is compacted in the lateral dimension when subjected to a uniaxial vertical stretch. Nevertheless, some materials expand counterintuitively in the lateral dimension under a uniaxial stretch, and are known as auxetic materials with a negative Poisson’s ratio (NPR).^18^ Instead of shrinking or expanding under uniaxial stretch, a few materials stay stationary in the lateral direction, and manifest themselves as a family of anepirretic materials with zero Poisson’s ratio (ZPR),^19^ such as natural cork and some artificial mechanical metamaterials with specially designed geometries.^20^ Natural cork was chosen empirically as a stopper before the concept of Poisson’s ratio was introduced, and the ZPR property made it easy to separate the stoppers from the bottle.^21^ According to their remarkable capacities of energy absorption and impact resistance, materials with ZPR are expected to have an array of practical applications in sonar, hydrophones, and telecommunication optical cables, where the dimensions of these devices need to be stable even in high-pressure environments such as in the deep ocean.^18^ Attributable to the unusual and compelling properties, anepirretic materials, including carbons, have been the subject of intense experimental and theoretical research.

Carbon allotropes usually exhibit PPR characteristics. The Poisson’s ratios of graphite, diamond, CNTs, and graphene have been demonstrated experimentally or theoretically to be ∼0.2,^22^ 0.069,^23^ 0.27 to 0.33 (depending on the tube diameter and chirality),^24^^,^^25^ and 0.16-0.186,^25^^,^^26^ respectively. Carbon materials with NPR or ZPR can be constructed experimentally and theoretically through the assembly of CNT or graphene.^27^ CNT sheets (*viz*. buckypaper) fabricated via the filtration of CNTs, display continuously tunable in-plane Poisson’s ratio from positive to negative and show anepirretic properties in some state.^28^^,^^29^ Graphene aerogel, a self-assemble of randomly oriented graphene oxide, displays reproducible near-ZPR in both compression directions under compression and release cycles in air/acetone,^30^ or a continuous change from PPR to NPR at different freezing temperatures.^27^ Furthermore, carbon allotropes based on graphene or CNT, such as two-dimensional ripple graphene,^31^ penta-graphene,^10^ and Me-graphene,^32^ have been simulated through state-of-the-art theoretical methods, and are predicted to reveal in-plane ZPR phenomenon because of the wrinkled layers acting like mechanical metamaterials.^18^ However, these carbons are assembled from *Van der Waals* interaction buckled CNTs or graphene, and as such they are soft and flexible. The design and synthesis of new carbon allotropes with both superhardness and ZPR is still a research gap and quite challenging.

Here, we theoretically report a new three-dimensional carbon allotrope established from covalently bonded CNT with anomalous wrinkled walls, named covalent carbon nanotube (CCN). The mechanical and thermal dynamic stabilities at ambient pressure were estimated from the calculated elastic constants and phonon dispersion spectra, respectively. Molecular dynamics simulations were conducted to test the structural stability at high temperature up to 1500 K. Mechanical and electrical properties, including Poisson’s ratio, hardness, tensile and compressive properties, and Young’s modulus, were predicted. Moreover, two B-C-N polymorphs CCN-C_8_N_12_ and CCN-B_12_N_8_, with superhardness and ZPR, are constructed by substituting boron and nitrogen for carbon atoms.

## Results and discussion

**Figure 1:**
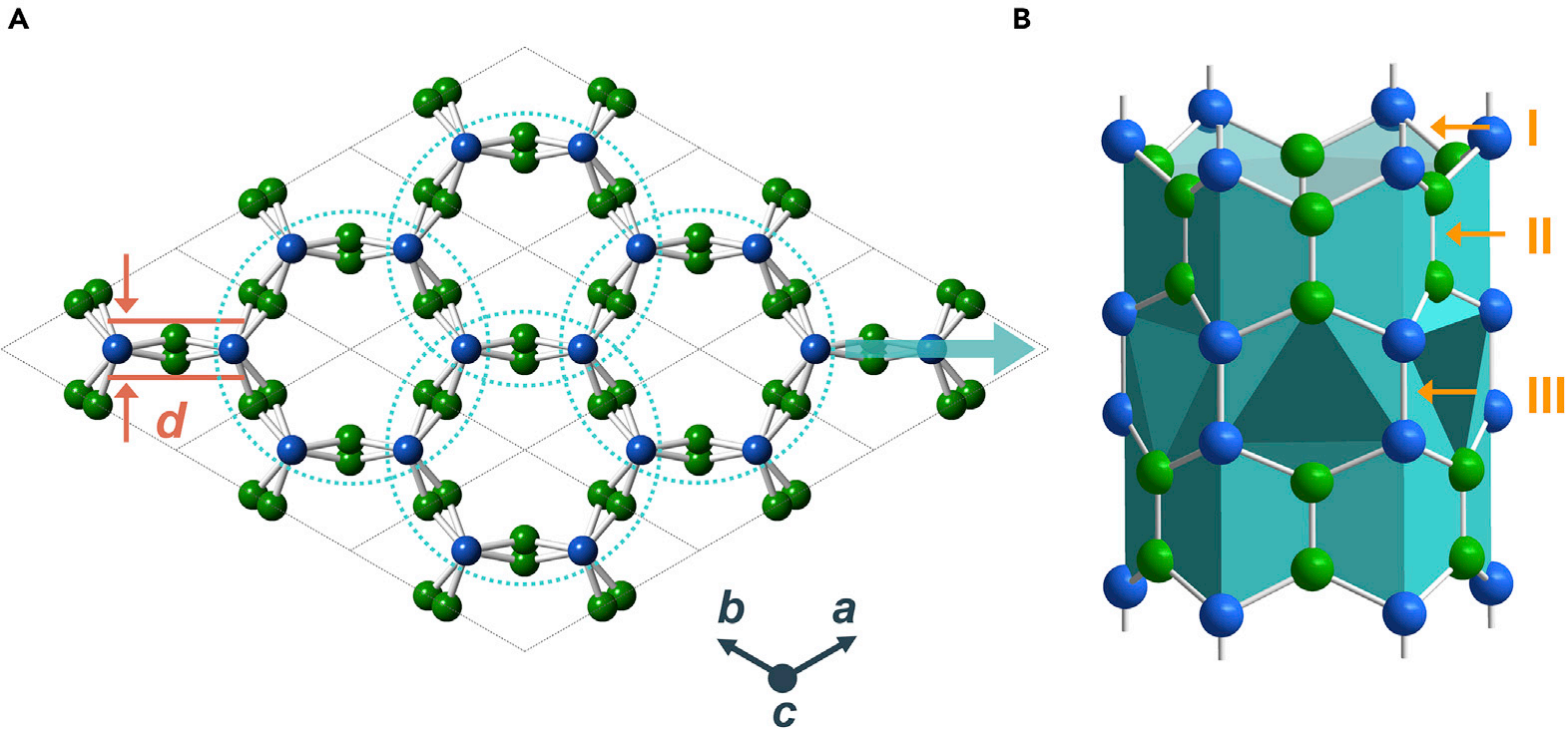
Crystal structure (A) Crystal structure of CCN viewed along the axial direction. Constructed CNT blocks with wrinkled walls are symbolled by dashed blue circles. (B) A nanotube building block of CCN. It is a deformed zigzag (6,0) single-walled CNT, with a wall thickness (*d*) of 0.506 Å. There are two inequivalent atoms, viz *sp*^3^-hybridized C1 (bule spheres) and *sp*^2^-hybridized C2 (green spheres), leading to three C-C bonds, labeled Bond-I, -II, and -III.

As shown in Figure 1, the CCN with hexagonal honeycomb configuration we proposed is theoretically fabricated from covalent buckling of wall-shared zigzag (6,0) CNTs. The crystal has a *P*63/*mcm* symmetry (space group number: 193) with a hexagonal lattice. The optimized lattice constants are *a* = *b* = 4.701 Å and *c* = 8.233 Å. There are two inequivalent atoms of C1 and C2, occupying the Wyckoff positions of 8*h* (0.6667, 0.3333, 0.5919) and 12*k* (0.4461, 0, 0.6693), respectively. The eight C1 atoms in a conventional cell are *sp*^3^ hybridized for interlocking the tubes, and the twelve C2 atoms inherit the *sp*^2^ hybridization of CNTs with a ratio of *sp*^2^:*sp*^3^ atoms of 3:2. There are three types of covalent bonds in CCN. Bond-I (*sp*^2^-*sp*^3^) is quasi-parallel to the lateral direction of the tube with a bond length of 1.520 Å. Bond-II (*sp*^3^-*sp*,^3^ 1.514 Å) and -III (*sp*^2^-*sp*,^2^ 1.328 Å) extend axially along the nanotube, with bond lengths smaller than that of diamond (1.54 Å) or graphite (1.42 Å). The top view of CCN along lattice *c* visually indicates that the walls of the zigzag (6,0) CNTs are winkled alternating concave and convex hexagonal interface, leading to a thick wall with *d* = 0.506 Å. Notably, CNT and graphene are common building blocks for constructing 3D carbon allotropes,^17^^,^^33^^,^^34^^,^^35^ while the nanotube walls or graphene sheets of the assembled 3D polymorphs are usually flat and a one-atom thick, which is totally different from CCN.

**Figure 2:**
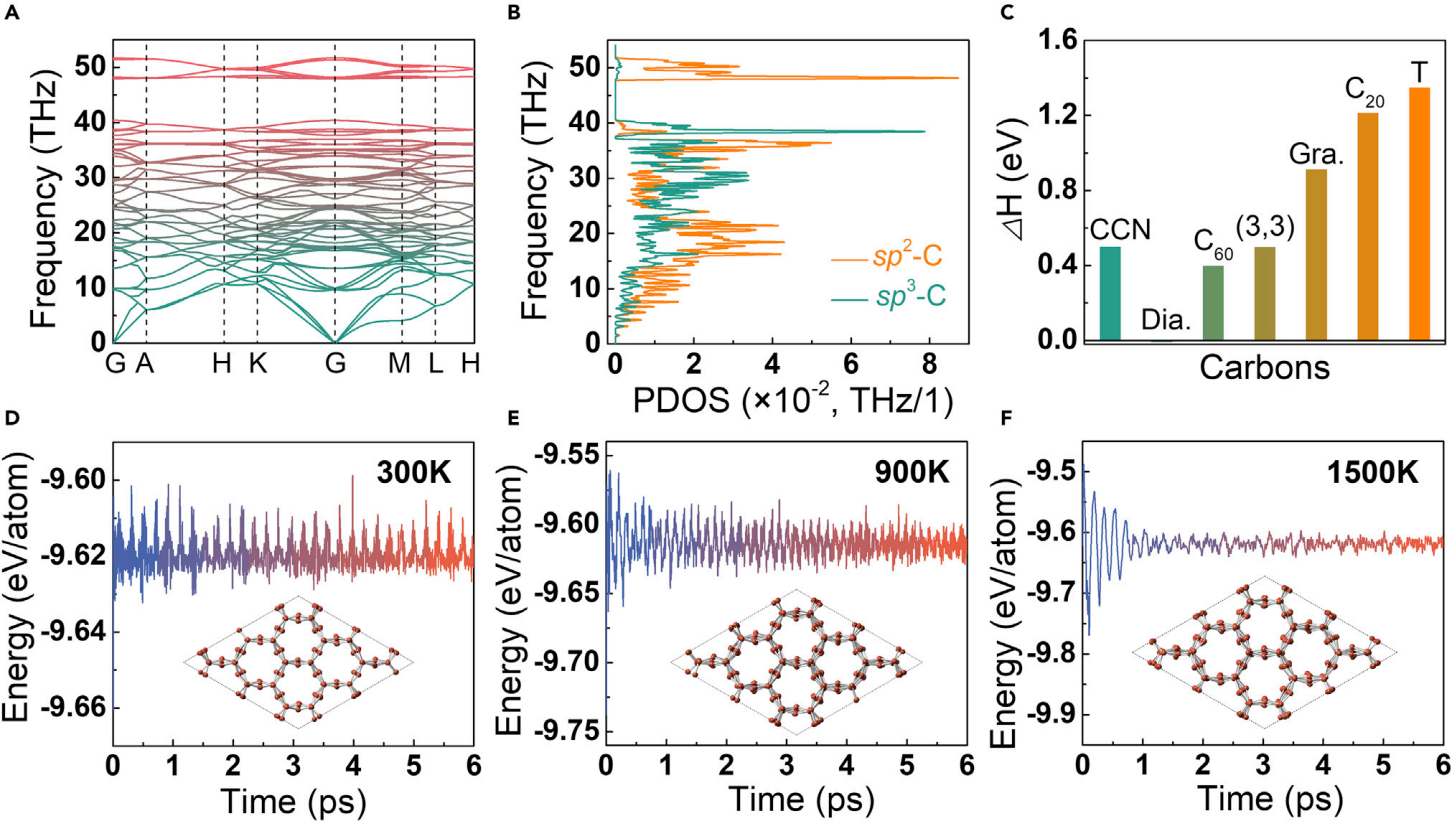
Crystal structure stability of CCN at ambient conditions and high temperatures (A) Calculated phonon dispersion spectra at ambient conditions. (B) Calculated phonon PDOS of *sp*^2^-C and *sp*^3^-C, respectively. (C) Relative enthalpies (*Δ*H) of CCN, natural diamond (Dia.), and experimentally synthesized carbons to graphite at ambient pressure. The calculated relative enthalpies of CCN, fullerene C_60_, (3,3) CNT, graphdiyne (Gra.), fullerene C_20_, and T-carbon are 0.50, 0.40, 0.50, 0.91, 1.21, and 1.35 eV/atom, respectively. (D–F) Potential energy of CCN at room temperature of 300 K, and at high temperatures of 900 K and 1500 K, respectively. The insets illustrate the final structures of CCN after AIMD relaxation at 300 K, 900 K, and 1500 K, respectively.

To investigate the structural stability of CCN, phonon dispersion spectra, phonon partial density of states (PDOS), and mechanical elastic constants were carried out at ambient conditions. Given the appearance of soft phonon modes in phonon spectra or PDOS may leading to structure distortion, it is critical to validate the dynamic stability of the crystal lattice vibrations by the phonon spectra and PDOS. As shown in Figures 2A and 2B, the absence of imaginary modes in the whole Brillouin zone confirms the dynamic stability of CCN at ambient conditions. The mechanical elastic constants were calculated to guarantee the mechanical stability of CCN. According to Born stability criteria,^36^ independent elastic constants *C*_ij_ of a 3D hexagonal lattice should comply with the following formula: *C*_11_ > |*C*_12_|, (*C*_11_+2*C*_12_)*C*_33_ > 2*C*_13_^2^, and *C*_44_ > 0. The calculated elastic constants of CCN are listed in Table 1. We find that all these criteria are satisfied, and thus this newly proposed carbon structure is mechanically stable.

**Table 1:** Mechanical properties of CCN, CCN-C_8_N_12_, CCN-B_12_N_8_, and diamond

Structure	CCN	CCN-C_8_N_12_	CCN-B_12_N_8_	Diamond
*C*_ij_	*C*_11_ = 365.8 *C*_12_ = 76.7 *C*_13_ = −1.8 *C*_33_ = 940.8 *C*_44_ = 262.9	*C*_11_ = 545.3 *C*_12_ = 88.4 *C*_13_ = 3.8 *C*_33_ = 1020.8 *C*_44_ = 222.6	*C*_11_ = 619.4 *C*_12_ = 151.4 *C*_13_ = 6.82 *C*_33_ = 808.7 *C*_44_ = 240.3	*C*_11_ = 1093.3 *C*_12_ = 133.8 *C*_44_ = 588.7
*B*	190.3	249.5	264.1	453.6
*G*	223.0	258.8	263.0	542.4
*E*_*a/b*_	349.7	530.9	582.3	1064.1
*E*_*c*_	940.8	1020.8	808.6	1064.1
*v*	0.08	0.11	0.13	0.07
*v*^Exp.^				0.069^23^
*k*	0.85			0.84
*H*_v_^Chen^	53.9	50.9	48.8	95.1
*H*_v_^Gao^	82.8			97.3
*H*_v_^Exp.^				96 ± 5^49^

Calculated elastic constants *C*_ij_ (GPa), bulk modulus *B* (GPa), shear modulus *G* (GPa), Young’s modulus along *a*- or *b*-axis (*E*_a/b_, GPa) and *c*-axis (*E*_c_, GPa), calculated Poisson’s ratio (*v*) and experimental Poisson’s ratio (*v*^Exp.^), *B*/*G* ratio (*k*), calculated Vickers hardness based on Chen’s (*H*_v_^Chen^, GPa) and Gao’s model (*H*_v_^Gao^, GPa), and experimental Vickers hardness (*H*_v_^Exp.^, GPa).

Total energy calculations of CCN and other theoretical and experimental carbons at 0 K were performed to investigate the thermodynamic stability of CCN, as shown in Figure 2C. Although CCN is metastable relative to graphite and diamond, it is energetically preferable or comparable to a number of polymorphs that have been experimentally synthesized, including crystal C_60_, graphdiyne,^1^ T-carbon,^37^ and the smallest CNT (3,3)^38^ and fullerene C_20_,^39^ clarifying their synthesis possibility. Herein we have performed AIMD simulations at room temperatures (300 K) and high temperatures up to 1500 K to guarantee the thermal stability of CCN upon heating. As shown in Figures 2D–2F, our calculations conclude that the vibration amplitude of the atoms near the equilibrium positions increases with increasing temperature and that the crystal maintains its original structure at a high temperature of at least 1500 K. In addition, the experimental synthesis of covalent nanotubes might light up the achievement of CCN.^40^ Recently, Koner et al designed a route for constructing one-, two-, and three-dimensional nanotubular covalent architectures based on the reversible aldehyde-amine condensation, and present the synthesis of a one-dimensional covalently bonded nanotubes by Schiff base reaction through taking tetratopic amine-functionalized triptycene and a linear dialdehyde as building blocks. Similarly, CCN is expected to be synthesized based on this ingeniously designed route with appropriate building blocks and reaction conditions.

The mechanical properties including Poisson’s ratio (*ν*), Vickers hardness (*H*_v_), bulk modulus (*B*), shear modulus (*G*), and Young’s moduli (*E*) are listed in Table 1. Poisson’s ratio is defined as *ν*_ij_ = -*ε*_j_/*ε*_i_, where *ε*_j_ is the lateral strain in *j*-direction and *ε*_i_ is the imposed longitudinal strain. Poisson’s ratio represents the elastic deformation behavior of a material under appropriate external force. For a polycrystalline, the Poisson’s ratio is calculated by formula *ν* = (3*B*-2*G*)/[2(3*B* + *G*)] based on the Voigte-Reusse-Hill approximation.^41^ The calculated Poisson’s ratio of polycrystalline CCN is 0.08, comparable to the experimental and theoretical values of diamond (*viz*. 0.069 and 0.07, respectively).^23^ However, the anisotropic Poisson’s ratio of single crystal CCN is very different from the isotropic one of the diamonds. Single crystal diamond possesses PPR performance in *x*-, *y*-, and *z*-axis with the same value of 0.109, while single crystal CCN shows ZPR in the direction depending on the CNT constructions. The corresponding anisotropic Poisson’s ratios of CCN in *x*-, *y*- and *z*-axis are *ν*_xy_ = *ν*_yx_ = 0.210, *ν*_xz_ = *ν*_yz_ = −0.002, *ν*_zx_ = *ν*_zy_ = −0.004, respectively. That is, the elastic deformation in *z*-axis (tube axial direction) rarely has an effect on the *x*- and *y*-axis (tube radial direction) under tension or compression, and vice versa.

**Figure 3:**
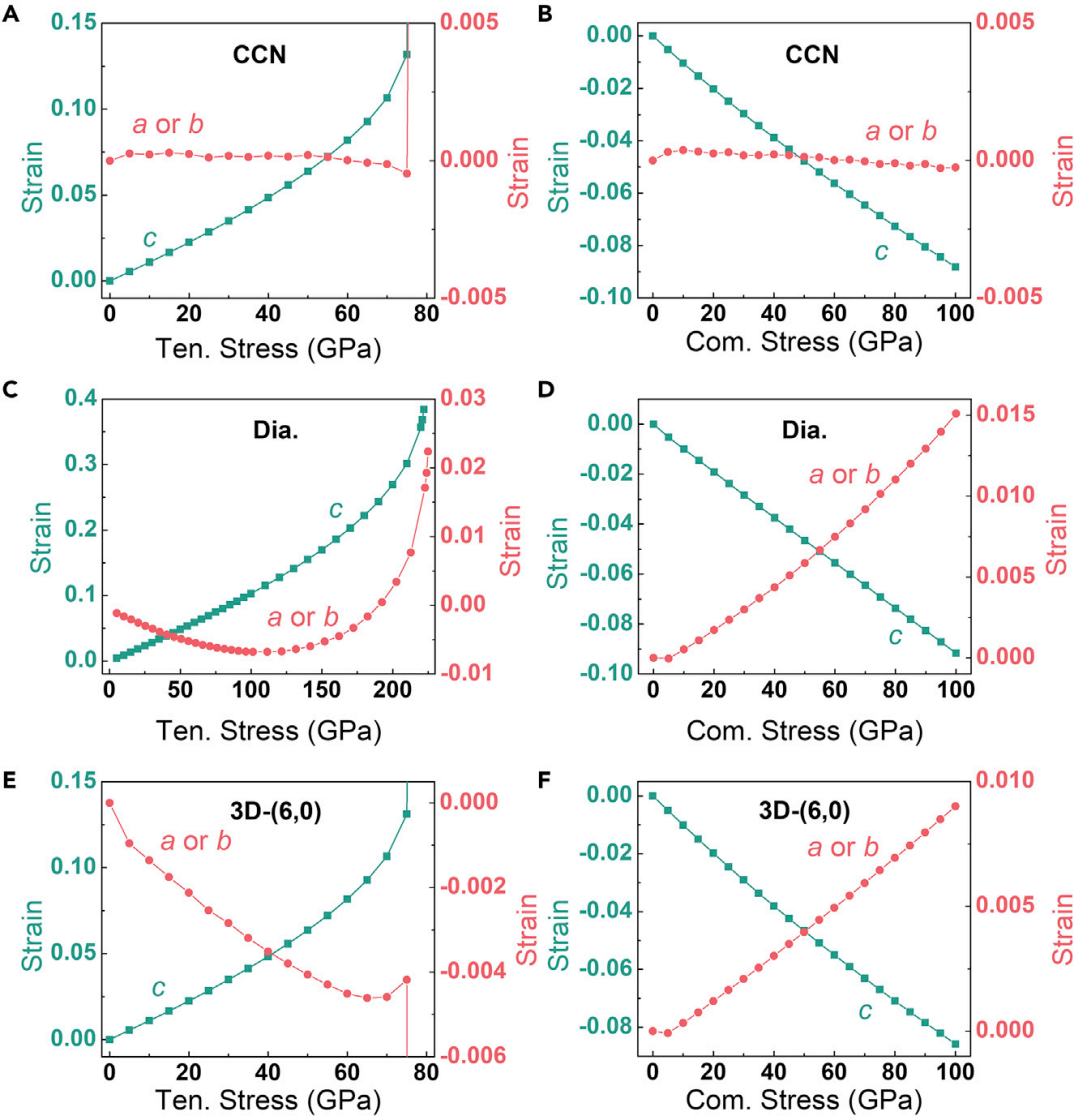
Mechanical properties of carbons (A–F) Lattice strains of CCN (A and B), diamond (C and D), and 3D-(6,0) (E and F) under uniaxial tensile (Ten.) and compressive (Com.) stress along the *c*-axis of the crystal.

Furthermore, we investigated the structural evolution of single crystal CCN under both tensile and compressive stresses at large structural strain (Figure 3), and diamond and 3D-(6,0) were also simulated as comparisons. For diamond, when lattice *c* is shortened (lengthened) under a *c*-axis compressive (tensile) stress, lattice *a* or *b* is raised under compact or shows a parabola-like trend under stretch. Nevertheless, lattice *a* and *b* of CCN are approximately invariant under both tensile and compressive stress along lattice *c* direction. When compressing CCN along *c*-axis up to 100 GPa, lattice *c* continues to shrink to 8.8%, while the *a*- or *b*-axis strain is almost zero (variation between 3.74 × 10^−4^ and −2.79 × 10^−4^). When extended in lattice *c* to fracture at the maximum stress of 76 GPa (*viz*. tensile strength) with strains exceeding 13%, the length of lattice *a* or *b* vibrates around the zero line (2.88 × 10^−4^ to −4.72 × 10^−4^). This anomalous structural evolution of CCN at large deformations is coincident with its ZPR characteristic at elastic deformation.

**Figure 4:**
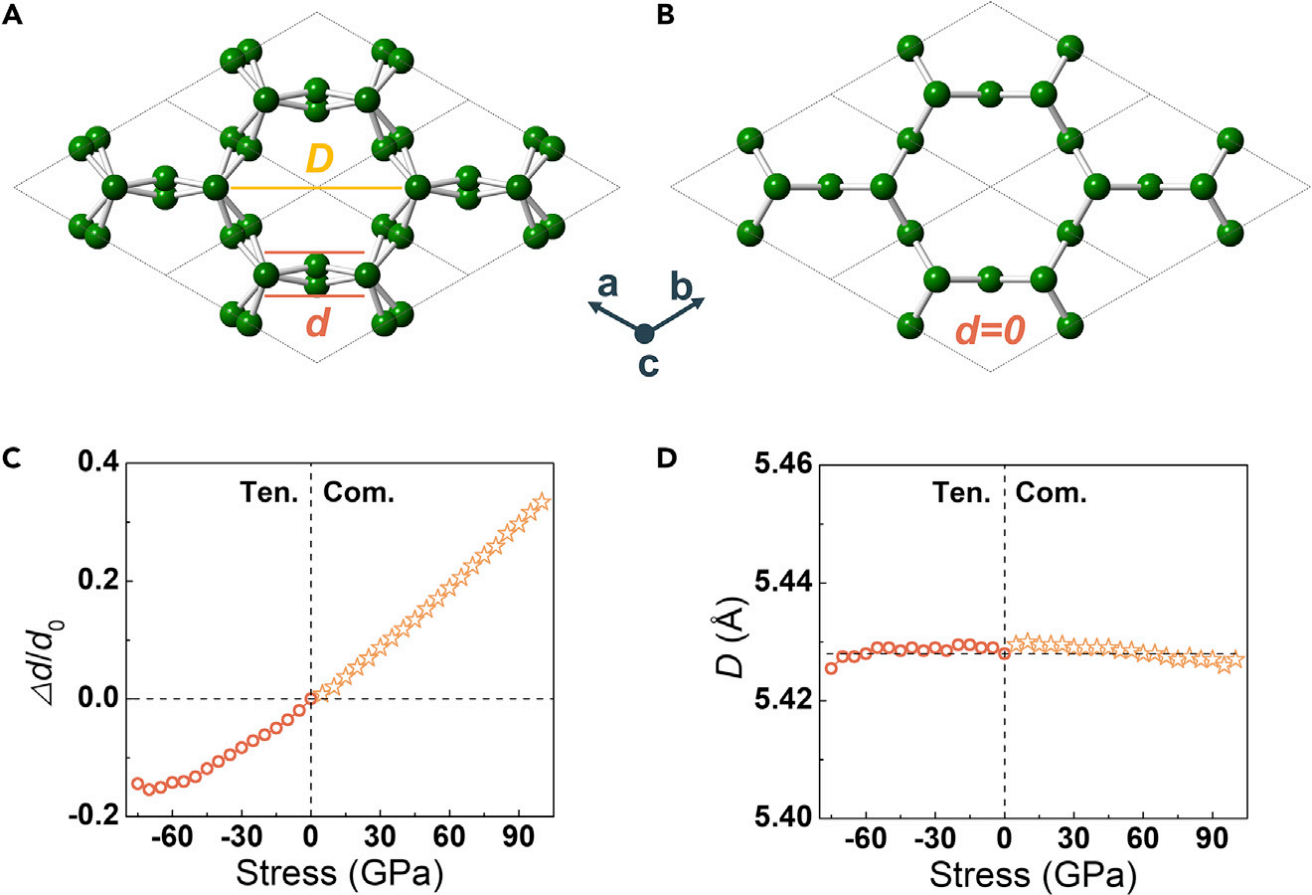
Structural evolution of carbons under tensile and compressive stress (A and B) Top views of CCN and 3D-(6,0) along lattice *c*, respectively. Symbol *d* is the tube wall thickness and *D* is the tube diameter. (C and D) Evolution of tube wall thickness (*d*) and diameter (D) for CCN under tensile (Ten.) and compress (Com.) stress along the *c*-axis. *Δd*/*d*_0_ is the strain of *d* under external force.

In order to obtain the source of axial ZPR performance and radial stability of CCN under large axial strain, the crystal structural deformation under both tensile and compressive stresses was detected in detail (Figure 4). It illustrates that the counterintuitive deformation behavior of CCN originates from the development of wall thickness. As shown in Figure 4C, the wall thickness (*d*) increases (decreases) to balance the internal stress when subjected to uniaxial shrinkage (stretching). As a result, the tube diameter (*D*) remains constant under the applied force, and the single crystal show an axial dimensional stability (Figure 4D). The structural evolution of 3D-(6,0) carbon^34^ is calculated to further demonstrate the effect of wrinkled nanotube walls. Similar to CCN, 3D-(6,0) carbon is also constructed from (6,0) CNTs, while its tube walls are flat (*d* = 0, Figure 4B). 3D-(6,0) possesses a positive axial Poisson’s ratio of *ν*_zx_ = *ν*_zy_ = 0.07, and shows radial shrinkage (or expanding) deformation under axial tensile (or compressive) stress (Figures 3E and 3F). Therefore, the wrinkled tube wall is responsible for the ZPR performance and radial stability of CCN under large axial strain. Notably, geometric deformation is a common approach for designing mechanical metamaterials with ZPR or NPR.^20^^,^^42^

**Figure 5:**
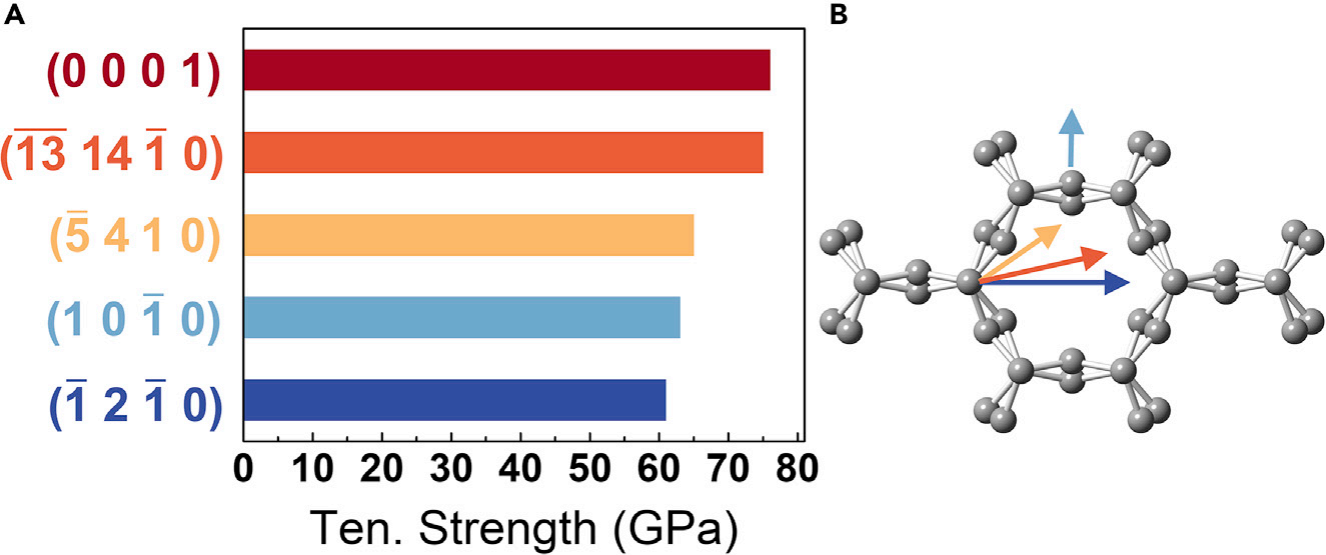
Tensile strength of CCN (A) Tensile (Ten.) strength of CCN under radial [crystal orientation ($\bar{1}$ 2 $\bar{1}$ 0), (1 0 $\bar{1}$ 0), ($\bar{5}$ 4 1 0), ($\bar{1}\bar{3}$ 14 $\bar{1}$ 0)] and axial [crystal orientation (0 0 0 1)] stress. (B) Radial stretching direction marked by corresponding-colored arrows.

The *sp*^2^ hybridization bonds are flexible, while the *sp*^3^ bonds are rigid. Therefore, CNTs with a tremendous axial Young’s modulus of ∼1 TPa^43^ are highly flexible and deformable in the radial directions, with radial Young’s modulus in the range of 0.3-4 GPa and 9.7-80.0 GPa for multiwalled CNTs.^44^^,^^45^ In contrast, the *sp*^3^ hybridized diamond is by far the hardest material. The covalently interlocked CCN inherits the superior stiffness of CNTs and exhibits a high axial Young’s modulus of 940.76 GPa, which is consistent with the modulus of CNTs. More importantly, the CCN obtains a well-developed radial Young’s modulus (349.75 GPa) from the intertubular covalent bonds, reaching several times or even hundreds of times that of the *Van der Waals* force assembled CNTs.^44^^,^^45^ Similarly, the calculated axial tensile strength of CCN is 76 GPa, which is half that of CNT^46^, while the radial tensile strength is significantly enhanced to 61-75 GPa originating from the covalent bonding (Figure 5). Both Gao’s and Chen’s model^47^^,^^48^ are adopted to evaluate the hardness (Table 1). The calculated Vickers hardness of diamond agrees with the experimental measurements,^49^ demonstrating the accuracy of the calculations. One dimensional pore structure in CCN results in a low density of 2.53 g/cm^3^, between graphite (2.23 g/cm³) and diamond (3.5 g/cm³). However, CCN is a superhard material with a Vickers hardness of 53.9-82.8 GPa, similar to *c*-BN (experimental: 63 ± 5 GPa,^49^ Gao’s model: 64.5 GPa^47^). On the other hand, the nanoporous structure of CCN presents a low bulk and shear modulus, about half of that of *c*-BN.^50^

**Figure 6:**
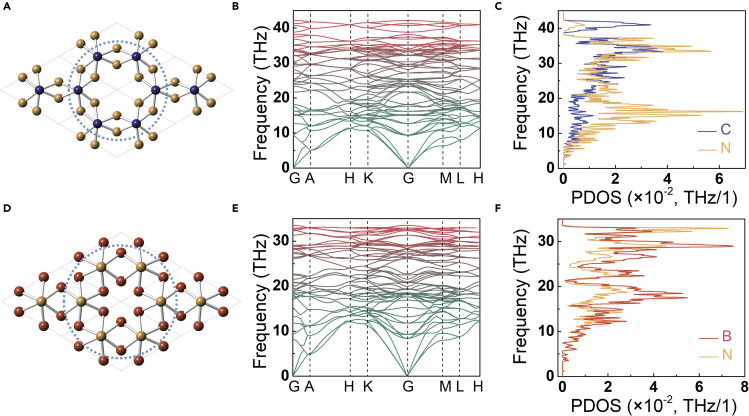
Crystal structures, phonon dispersion spectra, and phonon PDOS at ambient conditions (A) CCN-C_8_N_12_. Blue and yellow spheres represent C and N atoms, respectively. Lattice *a* = *b* = 4.367 Å, and *c* = 7.868 Å. (B and C) Calculated phonon spectra and PDOS of CCN-C_8_N_12_. No imaginary frequency in the whole Brillouin zones illustrates the thermal dynamic stability. (D) CCN-B_12_N_8_. Red and yellow spheres represent B and N atoms, respectively. Lattice *a* = *b* = 4.048 Å, and *c* = 8.859 Å. (E and F) Calculated phonon spectra and PDOS of CCN-B_12_N_8_. No imaginary frequency in the whole Brillouin zones illustrates the thermal dynamic stability.

As shown in Figure 1, there are two kinds of carbon atoms in CCN, including the *sp*^2^-hybridized carbons and the *sp*^3^-hybridized carbons. By replacing carbon with boron and nitrogen, seven polymorphs of CCN-B, CCN-B_8_C_12_, CCN-B_12_C_8_, CCN-B_8_N_12_, CCN-B_12_N_8_, CCN-C_8_N_12_, and CCN-C_12_N_8_ are constructed. Phonon spectra, phonon PDOS (Figure 6), and elastic constants (Table 1) confirm that only CCN-C_8_N_12_ and CCN-B_12_N_8_ are thermal dynamically and mechanically stable under ambient conditions. CCN-C_8_N_12_ and CCN-B_12_N_8_ (Figure 6) inherit the wrinkled tube walls of CCN, with the wall thickness of 1.056 Å and 1.668 Å, respectively, which are 2.1-3.3 times thicker than that of CCN. As a result of the wrinkled tube wall, the calculated anisotropic Poisson’s ratios of CCN-C_8_N_12_ and CCN-B_12_N_8_ in *z*-axis are *ν*_xz_ = *ν*_yz_ = 0.003 and *ν*_xz_ = *ν*_yz_ = 0.006, respectively, showing ZPR characteristics. The calculated Vickers hardness based on Chen’s model of CCN-C_8_N_12_ and CCN-B_12_N_8_ are 50.9 GPa and 48.8 GPa respectively, demonstrating their superhard properties.

**Figure 7:**
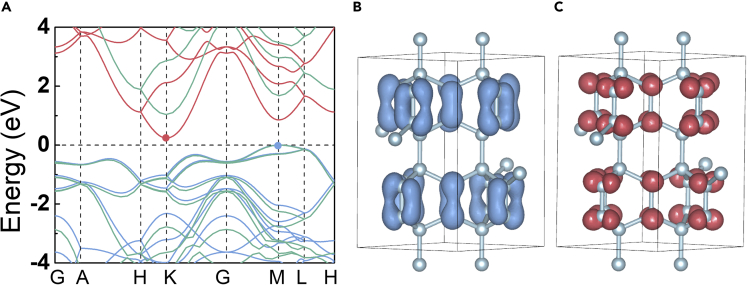
Electronic properties of CCN (A) Band structures calculated within LDA (red and blue lines) and HSE06 (green lines) functionals, respectively. (B) Charge distribution of VBM (*M* point) for CCN. (C) Charge distribution of CBM (*K* point) for CCN.

Based on the band structure of CCN at 0 GPa, we studied its electronic properties. As pictured in the band structure curves through both LDA and HSE06 functionals (Figure 7A), the valance band maximum (VBM) is located at the high-symmetry point *M* of Brillouin zones, while the conduction band minimum (CBM) is at *K* point, indicating that CCN is an indirect semiconductor with a narrow band gap (0.24 eV for LDA, and 1.23 eV for HSE06). The band decomposed charge densities of CCN at VBM and CBM are further shown to explain the origin of the electronic band gap (Figures 7B and 7C). The electrons of VBM and CBM are derived from *sp*^2^-hybridized C2 atoms without the contribution of C1 atoms and consist of π bonding and π^∗^ antibonding states, respectively. To conquer this gap, an electronic transition of the C2 atom from the lower π state to the higher π^∗^ state is a minimum requirement.

### Conclusions

A new *sp*^2^-*sp*^3^ hybridized carbon allotrope CCN with the hexagonal honeycomb crystal structure is proposed based on first principles calculations. The calculated phonon dispersion spectra and PDOS, elastic constant, and AIMD simulations confirm the dynamic, mechanical, and high temperature thermal stability of CCN. The relative enthalpies indicate that CCN is metastable compared to graphite and diamond, but it is energetically preferred to the synthesized CNT (3,3), fullerene C_20_, graphdiyne, and T-carbon. The wrinkled walls of nanotube blocks in CCN lead to anisotropic mechanical properties, especially the ZPR property in the axial dimension of the nanotubes, which are promising as an anepirretic material. Moreover, CCN remains the radial dimension even in the plastic deformation stage under large tensile or compressive strain. Electron band structure illustrates that CCN is semiconductive with a narrow indirect band gap. The proposed CCN with superhardness and ZPR performance has promising applications in engineering devices such as sonar, hydrophones, and telecommunication optical cables. Our study proposes a possible route for designing new three-dimensional carbon allotropes with both superhardness and ZPR properties.

### Limitations of the study

In this work, we investigated the covalent carbon nanotube and derived B-C-N polymorphs with superhardness and zero Poisson’s ratio (ZPR) based on theoretical calculations. Therefore, future experimental study on synthesizing this kind of materials and accurately characterizing the microstructure and mechanical properties is expected. Our study on B-C-N polymorphs proposed that covalent nanotube with wrinkled tube walls is superior with superhardness and ZPR, so it is promising to expand to boride, carbide, and nitride, which consist of covalent nanotube with wrinkled walls, for obtaining more materials with superhardness, ZPR, and other interesting physical properties.

## STAR★Methods

### Key resources table

**Table:** 

REAGENT or RESOURCE	SOURCE	IDENTIFIER
**Software and algorithms**		

CALYPSO	Wang et al., 2010	http://www.calypso.cn/
CASTEP	Segall et al., 2002	http://www.castep.org/
VASP	Kresse and Furthmüller, 1996	https://vasp.at/

### Resource availability

#### Lead contact

Further information and requests for resources should be directed to and will be fulfilled by the lead contact Meng Hu (humeng@ujs.edu.cn).

#### Materials availability

This study did not generate any unique reagents.

### Experimental model and subject details

Our study does not use experimental models typical in the life sciences.

### Methods details

All methods can be found in the supplemental information.

### Quantification and statistical analysis

Our study does not use quantification and statistical analysis.

## Data Availability

• Data reported in this paper will be shared by the lead contact upon request. • This paper does not report original code. • Any additional information required to reanalyze the data reported in this paper is available from the lead contact upon request.
