# Supplementary material for: Covalent three-dimensional carbon nanotube and derived B-C-N polymorphs with superhardness and zero Poisson’s ratio
Source: iScience. 2022 Nov 13;25(12):105563. doi: 10.1016/j.isci.2022.105563 (PMC9700005; doi:10.1016/j.isci.2022.105563)
Supplement: Document S1. Supplemental text [file mmc1.pdf]

iScience, Volume *25*

Supplemental information

Covalent three-dimensional carbon nanotube and derived B-C-N polymorphs with superhardness and zero Poisson's ratio

Shuang Chen, Meng Hu, Lingyu Liu, Yilong Pan, Penghui Li, Julong He, and Jianning Ding

Supplementary Text

METHODS DETAILS

Crystal structure prediction

Crystal Structure Analysis by Particle Swarm Optimization (CALYPSO) code[1] was used for seeking energetically favorable carbon allotropes with cell sizes of up to 24 atoms/cell and high pressures up to 50 GPa.

Physical properties calculations

The structural optimizations, elastic constants, Young's modulus, bulk modulus, shear modulus, Poisson's ratio, electron band structure, and phonon spectra and PDOS were conducted within Cambridge Sequential Total Energy Package (CASTEP)[2] based on density functional theory (DFT)[3,4]. The local density approximation (LDA) of Ceperley and Alder parameterized by Perdew and Zunger (CA-PZ)[5,6] was adopted for the exchange-correlation functional. The OTFG ultrasoft pseudopotential was employed to describe the electron-electron interaction[2] at a plane-wave energy cutoff of 440 eV. The Broyden-Fletcher-Goldfarb-Shanno (BFGS) method was used for the convergence criteria[7]. Structural optimization was completed with a convergence tolerance for energy, maximum force, maximum stress and maximum displacement no more than 5.0×10^{-6} eV/atom, 0.01 eV/Å, 0.02 GPa, and 5.0×10^{-4} Å, respectively. A *k*-point grid with separation of $2\pi\times0.07$ $Å^{-1}$ was generated using the Monkhorst-Pack grid parameters[8]. The finite displacement method was used to calculate the phonon frequency dispersion of CCN. Given LDA usually underestimating the band gap of a semiconductor, Heyd-Scuseria-Ernzerhof (HSE06) hybrid functional[9] embedded in CATEP was used to revise the band gap. The Norm-conserving pseudopotential[10] was employed to describe the electron-electron interaction. The calculated band gap of diamond within HSE06 functionals was 5.35 eV, very close to the experimentally observed 5.48 eV[11]. The elastic constants, bulk modulus, shear modulus and Young's modulus were calculated based on Voigt-Reuss-Hill approximation[12] using a primitive cell. The uniaxial tensile and compressive test were performed by means of fixed-stress method, whereby a fixed stress was gradually applied in the uniaxial direction and the whole lattice constants were optimized until the structural stress tensors were less than 0.02 GPa. As a comparison, the tensile strength of diamond along (100) was calculated to be 222 GPa, which is at the same level as the strain-fixed calculated results[13,14]. The charge density of the band decomposition was calculated using Vienna Ab initio Simulation Package (VASP)[15]. Based on canonical ensemble (NVT)[16], *ab initio* molecular dynamics (AIMD) simulations were performed from 300 K to 1500 K for 6 ps with a time step of 2 fs, and a 3×3×3 supercell of CCN with 540 atoms are applied.

REFERENCES

1. Wang, Y., Lv, J., Zhu, L., and Ma, Y. (2010). Crystal structure prediction via particle-swarm optimization. Phys. Rev. B *82*, 094116. https://doi.org/10.1103/PhysRevB.82.094116.
2. Segall, M.D., Lindan, P.J.D., Probert, M.J., Pickard, C.J., Hasnip, P.J., Clark, S.J., and Payne, M.C. (2002). First-principles simulation: Ideas, illustrations and the CASTEP code. J. Phys.: Condens. Mat. *14*, 2717-2744. https://doi.org/10.1088/0953-8984/14/11/301.
3. Hohenberg, P., and Kohn, W. (1964). Inhomogeneous electron gas. Phys. Rev. *136*, B864-B871. https://doi.org/10.1103/PhysRev.136.B864.
4. Kohn, W., and Sham, L.J. (1965). Self-consistent equations including exchange and correlation effects. Phys. Rev. *140*, 1133-1142. https://doi.org/10.1103/PhysRev.140.A1133.
5. Ceperley, D.M., and Alder, B.J. (1980). Ground state of the electron gas by a stochastic method. Phys. Rev. Lett. *45*, 566-569. https://doi.org/10.1103/PhysRevLett.45.566.
6. Perdew, J.P., and Zunger, A. (1981). Self-interaction correction to density-functional approximations for many-electron systems. Phys. Rev. B *23*, 5048-5079. https://doi.org/10.1103/PhysRevB.23.5048.
7. Pfrommer, B.G., Côté, M., Louie, S.G., and Cohen, M.L. (1997). Relaxation of crystals with the quasi-Newton method. J. Comput. Phys. *131*, 233-240. https://doi.org/10.1006/jcph.1996.5612.
8. Monkhorst, H.J., and Pack, J.D. (1976). Special points for Brillouin-zone integrations. Phys. Rev. B *13*, 5188-5192. https://doi.org/10.1103/PhysRevB.13.5188.
9. Heyd, J., Scuseria, G.E., and Ernzerhof, M. (2003). Hybrid functionals based on a screened Coulomb potential. The Journal of Chemical Physics *118*, 8207-8215. https://doi.org/10.1063/1.1564060.
10. Lin, J.S., Qteish, A., Payne, M.C., and Heine, V. (1993). Optimized and transferable nonlocal separable ab initio pseudopotentials. Phys. Rev. B *47*, 4174-4180. https://doi.org/10.1103/PhysRevB.47.4174.

11. Gali, A., Janzen, E., Deak, P., Kresse, G., and Kaxiras, E. (2009). Theory of spin-conserving excitation of the N-V$^-$ center in diamond. Phys. Rev. Lett. *103*, 186404. https://doi.org/10.1103/PhysRevLett.103.186404.
12. Hill, R. (1952). The elastic behaviour of a crystalline aggregate. Proc. Phys. Soc. A *65*, 349-354. https://doi.org/10.1088/0370-1298/65/5/307.
13. Zhang, Y., Sun, H., and Chen, C. (2006). Structural deformation, strength, and instability of cubic BN compared to diamond: A first-principles study. Phys. Rev. B *73*, 144115 https://doi.org/10.1103/PhysRevB.73.144115.
14. Telling, R.H., Pickard, C.J., Payne, M.C., and Field, J.E. (2000). Theoretical strength and cleavage of diamond. Phys. Rev. Lett. *84*, 5160-5163. https://doi.org/10.1103/PHYSREVLETT.84.5160.
15. Kresse, G., and Furthmüller, J. (1996). Efficient iterative schemes for ab initio total-energy calculations using a plane-wave basis set. Phys. Rev. B *54*, 11169-11186. https://doi.org/10.1103/PhysRevB.54.11169.
16. Nosé, S. (1984). A unified formulation of the constant temperature molecular dynamics methods. J. Chem. Phys. *81*, 511-519. https://doi.org/10.1063/1.447334.
